# Pooling isolates to address the diversity in antimicrobial susceptibility of *Pseudomonas aeruginosa* in cystic fibrosis

**DOI:** 10.1128/spectrum.00449-23

**Published:** 2023-11-20

**Authors:** Sara Van den Bossche, Emmanuel Abatih, Lucia Grassi, Emma De Broe, Petra Rigole, Jerina Boelens, Joris Van Caenegem, Bruno Verhasselt, Iris Janssens, Eva Van Braeckel, Nick Versmessen, Piet Cools, Tom Coenye, Aurélie Crabbé

**Affiliations:** 1 Laboratory of Pharmaceutical Microbiology, Ghent University, Ghent, Belgium; 2 Data Analysis and Statistical Science (DASS), Ghent University, Ghent, Belgium; 3 Laboratory of Medical Microbiology, Ghent University Hospital, Ghent, Belgium; 4 Department of Diagnostic Sciences, Ghent University, Ghent, Belgium; 5 Department of Respiratory Medicine, Cystic Fibrosis Reference Centre, Ghent University Hospital, Ghent, Belgium; 6 Department of Internal Medicine and Pediatrics, Ghent University, Ghent, Belgium; 7 Center for Inflammation Research, Flemish Institute for Biotechnology, Ghent, Belgium; Weill Cornell Medicine, New York, New York, USA

**Keywords:** antibiotic susceptibility testing, *Pseudomonas aeruginosa*, diversity, minimal inhibitory concentration, pooling isolates, cystic fibrosis

## Abstract

The intrasample diversity in antibiotic resistance of *Pseudomonas aeruginosa* in people with cystic fibrosis (pwCF) may contribute to the low predictive value of antibiotic susceptibility testing (AST). Since often only few isolates per AST are included, a possible solution to decrease the diversity is pooling isolates, yet a comprehensive study on this approach is lacking. This study aims to characterize the intrasample diversity in antibiotic resistance of *P. aeruginosa* and to assess if pooling isolates could lower diversity. In 15 pwCF, 30 *P*. *aeruginosa* isolates were collected per patient sputum sample, and the minimal inhibitory concentration (MIC) was determined for three antibiotics commonly used to treat cystic fibrosis patients to evaluate intrasample diversity. Next, the minimal number of isolates necessary to be pooled for a consistent MIC was determined. Finally, we assessed if the MIC result of pooled versus single isolates could influence clinical interpretation. Intrasample MIC diversity was observed in most samples, with the highest frequencies for aztreonam and ceftazidime. Pooling isolates decreased diversity, and for each sputum sample, a consistent MIC was obtained after pooling two to nine isolates. The minimal number of pooled isolates for consistent MIC positively correlated with MIC diversity in single isolate testing. MIC diversity and categorical disagreement were correlated, and the level of categorical disagreement decreased in most samples when pooling nine isolates. Also, pooling isolates did not decrease the frequency of resistance detection in most samples. In conclusion, intrasample MIC diversity clearly impacts AST outcome for *P. aeruginosa* in pwCF, and pooling isolates is a promising approach to lower diversity.

**IMPORTANCE:**

People with cystic fibrosis (pwCF) often suffer from chronic lung infections with *Pseudomonas aeruginosa*. While antibiotics are still commonly used to treat *P. aeruginosa* infections, there is a high discordance between *in vitro* and *in vivo* antibiotic efficacy, which contributes to suboptimal antibiotic therapy. In the present study, we found that isolates from the same sputum sample had highly diverse antibiotic resistance profiles [based on the minimal inhibitory concentration (MIC)], which may explain the reported discrepancy between *in vitro* and *in vivo* antibiotic efficacy. Through systematic analysis, we report that pooling nine isolates per sputum sample significantly decreased intrasample diversity in MIC and influenced clinical interpretation of antibiotic susceptibility tests compared to single isolate testing. Hence, pooling of isolates may offer a solution to obtain a consistent MIC test result and could lead to optimizing antibiotic therapy in pwCF and other infectious diseases where diversity in antibiotic resistance is observed.

## INTRODUCTION

Chronic lung infections with *Pseudomonas aeruginosa* are still one of the most important causes of morbidity and mortality in people with cystic fibrosis (pwCF) (1). While antibiotics are the standard of care to treat *P. aeruginosa* lung infections in the cystic fibrosis (CF) population, clinical improvement is often not achieved (2). Since conventional antibiotic susceptibility testing (AST) poorly predicts antibiotic efficacy *in vivo* in the CF population (2, 3), antibiotic therapy is usually empiric and/or based on previous treatments of the patient, instead of on *in vitro* test results (4).

A possible explanation for the discrepancy between *in vitro* and *in vivo* antibiotic susceptibility of *P. aeruginosa* in chronically infected pwCF is the intrasample diversity in antibiotic resistance of isolates (5 – 8), combined with the fact that AST is only performed on a few sputum isolates, with high between-center variability in how many isolates are picked (3). Indeed, previous studies on phenotypic diversity of *P. aeruginosa* from one patient reported multiple susceptibility profiles per sample based on classification into susceptibility categories (5, 9 – 11). This phenotypic diversity is likely due to the genetic adaptation of *P. aeruginosa* to spatially segregated niches in the CF airways during chronic infection, characterized by varying local environmental conditions (such as oxygen levels and iron concentrations) (7, 8). Genetic adaptation results in a changing composition of the population over time and in mixed communities, wherein different phenotypes of *P. aeruginosa* interact. This interaction has, for example, been shown to result in higher resistance to the antimicrobial peptide LL-37 (12).

Many clinical laboratories select isolates for AST based on colony morphology, but these morphotypes were found to be poor predictors of antibiotic susceptibility in CF patients based on several studies (5, 13). Pooling four isolates per patient sample for AST was investigated as an alternative but failed to detect all resistant isolates (13).

A recent study by Rojas et al. investigating heterogeneity through phylogenetic reconstruction again highlights that profiling a single isolate for clinical reporting could lead to ineffective treatment and that there is high need for a strategy to perform AST on diverse *P. aeruginosa* populations (14). The present study aims at filling this gap by evaluating diversity in antibiotic resistance of *P. aeruginosa* for antibiotics commonly used to treat infections in pwCF and by systematically assessing the influence of pooling isolates on AST outcome.

## MATERIALS AND METHODS

**Fig 1:**
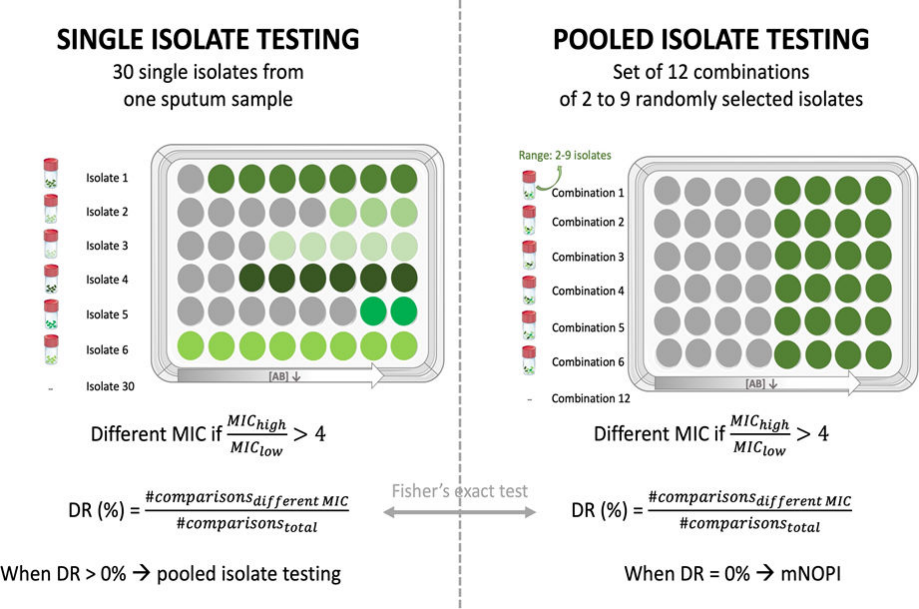
Schematic overview of the workflow in the present study. First, the intrasample MIC diversity was determined by testing the MIC for each isolate separately. If MIC diversity (shown in the left picture as growth inhibition at different antibiotic concentrations) was observed in single isolate testing, the MIC was determined for combinations of an increasing number of pooled isolates to assess the influence of pooling isolates systematically. For each number of pooled isolates (ranging from 2 to 9 isolates), the MIC was determined for 12 combinations of randomly selected isolates, and the number of pooled isolates was further increased until the diversity ratio became zero (shown in the right picture as growth inhibition for all 12 combinations at the same antibiotic concentration). For abbreviations and definitions, see Table 1.

The terminology specific to this study is described in Table 1, and a schematic overview of the workflow is depicted in Fig. 1.

**TABLE 1:** Terminology

Term	Description
MIC profile	MIC values obtained with 30 single isolates from one sputum sample for one antibiotic.
Set of 12 combinations	A set of 12 combinations of a particular number of pooled isolates randomly selected from 30 isolates from one sputum sample.
Number of pooled isolates (NOPI)	The number of isolates pooled in every combination of a set of 12 combinations. The number of pooled isolates ranges from 2 to 9.
MIC ratio (MR)	The MIC ratio is the ratio of the highest MIC to the lowest MIC when two single isolates or two combinations are compared.
Different MIC	The MIC of two single isolates or combinations is considered different if the MIC ratio is higher than 4. This definition originates from observing no biological and/or technical replicates for which the MIC ratio was higher than 4.
Diversity ratio (DR)	The diversity ratio is the ratio of the number of unique pairwise comparisons resulting in a different MIC value (i.e., MIC ratio >4) to the total number of unique pairwise comparisons. It was calculated for single isolate testing in every sputum sample (30 isolates) and for every set of 12 random combinations of isolates.
MIC diversity	The term MIC diversity is used if at least one pairwise comparison shows an MIC ratio >4, i.e., when diversity ratio >0%.
Consistent MIC	A consistent MIC is reached when the diversity ratio is 0% in a set of 12 combinations.
mNOPI	The minimal number of pooled isolates necessary to obtain a consistent MIC.
Categorical disagreement (CD)	Categorical disagreement (CD) is when the MIC determination puts two single isolates from the same sputum sample or two combinations from a set in a different EUCAST susceptibility category.
%CD	The percentage of unique pairwise comparisons resulting in categorical disagreement.

### Patient characteristics and sputum samples

Spontaneously expectorated sputum samples were collected from 15 pwCF chronically infected with *P. aeruginosa*, attending the outpatient clinic of Ghent University Hospital for routine follow-up. This study was approved by the ethics committee of Ghent University Hospital (registration number B670201836204). Only patients able to produce sputum, older than 12 years, chronically infected with *P. aeruginosa* (based on *P. aeruginosa*-positive culture in >50% of samples taken in the previous 12 months), and clinically stable at the time of sampling, were included. The following patient characteristics were extracted from patient records: sex, age, genotype, forced expiratory volume in 1 second predicted (FEV_1_) (as measured by spirometry) at the time of sampling, pancreatic insufficiency, infection time with *P. aeruginosa* (i.e., time since the first *P. aeruginosa*-positive culture), other isolated microorganisms up to 1 year before sampling, antibiotic use at time of sampling, and antimicrobial therapies in the year preceding the sampling.

### Isolation, identification, and AST of *P. aeruginosa*

Sputum samples were washed with sterile physiological saline to remove saliva (15). Afterward, samples were liquefied by adding an equal volume of sterile Sputasol (Oxoid) followed by shaking (250 rpm) at 37°C until the samples were liquid (30–60 min), following the manufacturer’s recommendations. Serial dilutions (10-fold) of the liquefied samples were plated on McConkey agar (Becton Dickinson) (100-µL full spread per plate) to enable selective growth of Gram negatives. Plates were incubated for 48 h at 37°C under normoxic conditions. For each sample, 35 lactose-negative single colonies were randomly selected and plated on Luria-Bertani (LB) agar (Lab-M), similarly as for previous studies where phenotypic diversity of *P. aeruginosa* in CF sputum was evaluated (9 – 11). Each isolate was identified by matrix assisted laser desorption/ionization (MALDI-TOF) mass spectrometry (Bruker) (16), and for each sputum sample, at least 30 isolates identified as *P. aeruginosa*. Mucoid isolates were found in all but one patient sample (i.e., patient 3). *P. aeruginosa* isolates were grown in LB broth (Lab-M) for 16–18 h (250 rpm, 37°C) for all subsequent experiments. The minimal inhibitory concentration (MIC) was determined by broth microdilution as described in ISO technical recommendations (ISO 20776-2:2021) for tobramycin (TCI Europe) (concentration range, 0–512 µg/mL), ceftazidime (Sigma-Aldrich) (concentration range, 0–8192 µg/mL), and aztreonam (TCI Europe) (concentration range, 0–4096 µg/mL). These three antibiotics are commonly used to treat respiratory infections caused by *P. aeruginosa* in pwCF (guidelines of the CF Trust, ECFS, CF Foundation) (17, 18). Briefly, for single isolate testing, bacteria were suspended in Mueller-Hinton broth (LabM) at a density of 5 × 10^5^ CFU/mL (as determined by OD_590_ measurement) and exposed to a twofold dilution series of antibiotics in a 96-well plate, and plates were incubated (18–20 h, 37°C). For pooled isolate testing, mixtures containing equal CFU for each isolate were made with a constant inoculum density of 5 × 10^5^ CFU/mL in Mueller-Hinton broth. After incubation, bacterial growth and the inhibition thereof were assessed measuring optical density at 590 nm using a plate reader (Perkin Elmer). The MIC was defined as the antibiotic concentration that resulted in at least 90% growth inhibition as compared to an untreated control.

### Systematic assessment of the influence of pooling isolates on antibiotic resistance

First, for each sputum sample, the intrasample MIC diversity was determined based on the MIC ratio and the diversity ratio (DR). To this end, the MIC was obtained for 30 single *P. aeruginosa* isolates per sample (Fig. 1), and this value (or median MIC value in case of triplicate) was compared pairwise between all single isolates in one sputum sample (*n* = 30). The MIC ratio is the ratio between the highest observed MIC (MIC_high_) and lowest observed MIC (MIC_low_) of the pair. The MIC of each pair of isolates was defined as different if the MIC ratio of two isolates was higher than 4. This threshold is based on EUCAST guidelines, as a twofold dilution in either direction is considered within the margin of error, considering biological and technical variability. The diversity ratio was defined as the percentage of unique pairwise comparisons resulting in a different MIC value (i.e., MIC_high_/MIC_low_ >4) to the total number of unique pairwise comparisons. The value for the diversity ratio can vary between 0% (i.e., for none of the comparisons the MIC was different) and 100% (i.e., for all possible comparisons the MIC was different). MIC profiles were considered diverse if the diversity ratio was greater than 0%. Both diversity ratio and MIC ratio are important characteristics of an MIC profile, as the diversity ratio is a measurement of how many isolates differ from each other in terms of MIC, while the MIC ratio represents the magnitude of the difference in MIC. Moreover, a diversity ratio of 0% means that no MIC diversity is observed, and thus, a consistent MIC is obtained.

If intrasample MIC diversity was observed in single isolate testing (i.e., if DR was higher than zero), the effect of pooling isolates on MIC was determined in a systematic way (Fig. 1). To this end, the MIC was determined for combinations of an increasing number of pooled isolates. For each number of pooled isolates (ranging from 2 to 9 isolates), the MIC was determined for 12 combinations of randomly selected isolates (i.e., a set of 12 combinations), by use of the function Random or RAND in Microsoft Excel. The number of pooled isolates was progressively increased for each antibiotic separately until a consistent MIC was obtained, i.e., the same MIC value regardless of which isolates are randomly picked for MIC testing. This number of pooled isolates was defined as the minimal number of pooled isolates necessary to obtain consistent MIC. To determine whether a consistent MIC could be obtained, the MIC was compared pairwise between all random combinations of one set of 12 combinations (*n* = 12) and was defined as different if the MIC ratio of two combinations was higher than 4 (considering technical/biological variability). Based on this definition of different MIC values, a diversity ratio was calculated for every set of 12 combinations.

### Data analysis

Experiments on isolates from patients 1 to 5 were performed in biological triplicate, while isolates of patient 6 to 15 were tested once, in accordance with EUCAST guidelines.

For each sputum sample, the MIC of single isolates was determined, and if diversity in MIC for an antibiotic was observed, the effect of pooling isolates was determined by assessing the diversity ratio for 12 random combinations of increasing numbers of pooled isolates. The diversity ratio of each set of 12 combinations was then compared with the diversity ratio of single isolate testing, using Fisher’s exact test, allowing to detect significant differences between results obtained with single isolate and pooled isolates testing. A Benjamini-Hochberg correction was performed to correct for multiple testing (19). The number of pooled isolates was progressively increased until a consistent MIC was obtained (i.e., diversity ratio was 0%). This number of pooled isolates was defined as the minimal number of pooled isolates necessary to obtain consistent MIC.

The correlation between continuous patient variables and the diversity ratio for aztreonam, ceftazidime, and tobramycin and the correlation between the diversity ratio of single isolate testing and the minimal number of pooled isolates for consistent MIC (mNOPI) for MIC profiles with diversity were determined using a Spearman’s rank order correlation. In all cases, assumptions of continuous or ordinal data and monotonicity were checked (20).

To determine the influence of pooling isolates on clinical interpretation, the percentage of categorical disagreement (%CD) was calculated for single isolates or a set of 12 combinations by comparing pairwise the susceptibility classification of single isolates or combinations. The %CD was defined as the percentage of unique pairwise comparisons resulting in a different susceptibility classification [based on EUCAST clinical breakpoints (v 12.0): tobramycin (S, susceptible ≤2 µg/mL; R, resistant >2 µg/mL], aztreonam (I, susceptible increased exposure ≤16 µg/mL, R >16 µg/mL) and ceftazidime (I ≤8 µg/mL, R >8 µg/mL)], from the total number of unique pairwise comparisons. A Pearson chi-square analysis was used to determine the odds ratio of encountering categorical disagreement (CD) in diverse samples compared to non-diverse samples. The %CD was compared between single isolate and pooled isolate testing using Fisher’s exact test. A Benjamini-Hochberg correction was performed to correct for multiple testing (19).

Data analysis was performed using R statistical software (version 1.4.1106) and SPSS statistics (version 24).

## RESULTS

### Patient characteristics

Fifteen sputum samples were collected from individual patients (aged 18–59 years), among which seven patients were homozygous for the *Phe508del* mutation, five patients were compound heterozygous for *Phe508del*, and three patients had two other CF-causing mutations (Table S1). All the study subjects suffered from pancreatic insufficiency, and the mean FEV_1_ was 58% predicted. Five out of 15 patients received cystic fibrosis transmembrane conductance regulator (CFTR)-modulator treatment at the time of sampling, and 12 patients were cyclically or chronically treated with inhaled antibiotics, among which eight actively at the time of sampling. Nine patients were on chronic oral macrolide treatment; other systemic antibiotics, such as *P. aeruginosa* eradication regimens, are listed in Table S1. In the year preceding the sampling, eight patients were co-infected with *Staphylococcus aureus*, and two patients, with *Achromobacter xylosoxidans* (Table S1).

### Intrasample MIC diversity for at least one antibiotic is found in a majority of sputum samples

**Fig 2:**
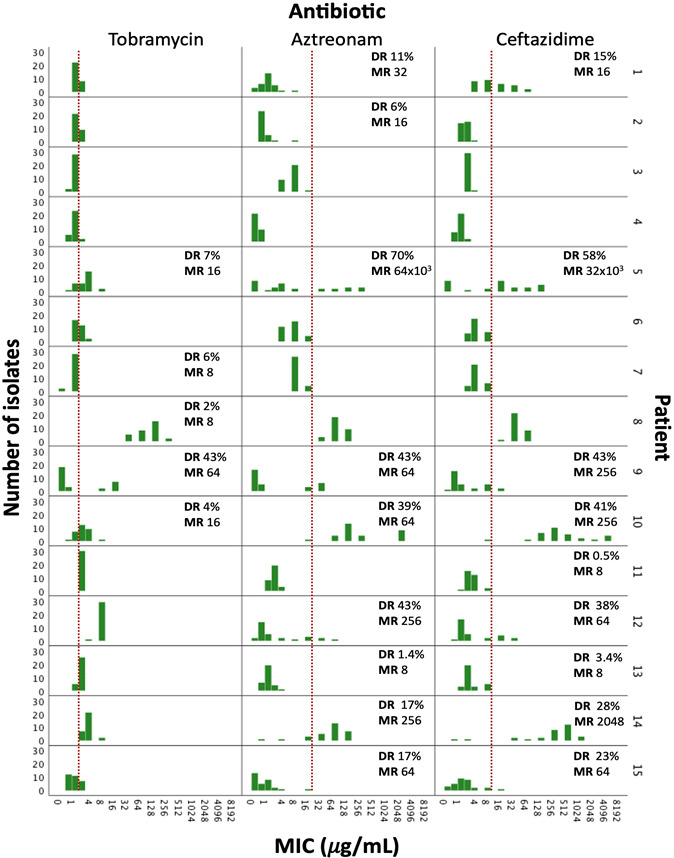
MIC profiles for 30 single isolates per CF patient sputum sample. On the horizontal axis, the MIC is shown; on the vertical axis, the frequency (i.e., number of isolates with that particular MIC) is shown for all 15 patients (patient number shown on the right). Results are shown for tobramycin, aztreonam, and ceftazidime. The DR, a measurement of how many isolates have a different MIC, and the MIC ratio (MR) between the lowest and the highest observed MIC value, indicating the magnitude of MIC difference, in the sputum sample, are included for samples with diversity. Clinical breakpoints according to EUCAST (v 12.0), which are within the range of the observed MIC values, are depicted in red for tobramycin (S ≤2 µg/mL, R >2 µg/mL), aztreonam (I ≤16 µg/mL, R >16 µg/mL), and ceftazidime (I ≤8 µg/mL, R >8 µg/mL).

Per sputum sample, 30 *P*. *aeruginosa* isolates were randomly selected, and the intrasample MIC diversity was determined for three antibiotics commonly used to treat CF patients. We chose to define intrasample diversity based on MIC values, rather than on categorization in susceptibility categories (5, 9 – 11), to make data interpretation independent of current clinical breakpoints, which are not adjusted to the CF patient population (21). The MIC profiles for aztreonam, ceftazidime, and tobramycin for each sample are shown in Fig. 2. Eighty percent of the sputum samples (12/15) showed intrasample MIC diversity for at least one antibiotic and a high variability in diversity ratio, and MIC ratio was observed between patients and antibiotics (range diversity ratio, 0.5%–70%; range MIC ratio, 8–64,000). MIC diversity was most frequently observed for aztreonam and ceftazidime (in both cases 9/15 samples; 60%) and less frequently for tobramycin (5/15 samples; 33%). The observed diversity ratio and MIC ratio were higher for aztreonam (median diversity ratio, 17%; median MIC ratio, 64) and ceftazidime (median diversity ratio, 28%; median MIC ratio, 64) compared to tobramycin (median diversity ratio, 6%; median MIC ratio, 16). In 3/15 samples, MIC diversity was observed for tobramycin, aztreonam, and ceftazidime simultaneously, while in 8/15 samples, diversity in MIC was observed for both aztreonam and ceftazidime.

### Intrasample MIC diversity can be linked to infection time with *P. aeruginosa*

Next, we assessed if MIC diversity was linked to patient characteristics. The correlation between the diversity ratio for aztreonam, ceftazidime, and tobramycin (Fig. 2) and all continuous patient variables (age, FEV_1_, infection time; Table S1) was calculated. Significant positive correlations were found between the diversity ratio for aztreonam (ρ = 0.780; *P* = 0.001) and ceftazidime (ρ = 0.819; *P* < 0.001) and the infection time with *P. aeruginosa* (ranging from 1 year to over 19 years; Table S1). No meaningful correlation analysis between the diversity ratio and antibiotic regimen was possible, due to the limited number of patient samples and the cross-sectional design of the study.

### Pooling isolates lowers intrasample MIC diversity and leads to a consistent MIC

**Fig 3:**
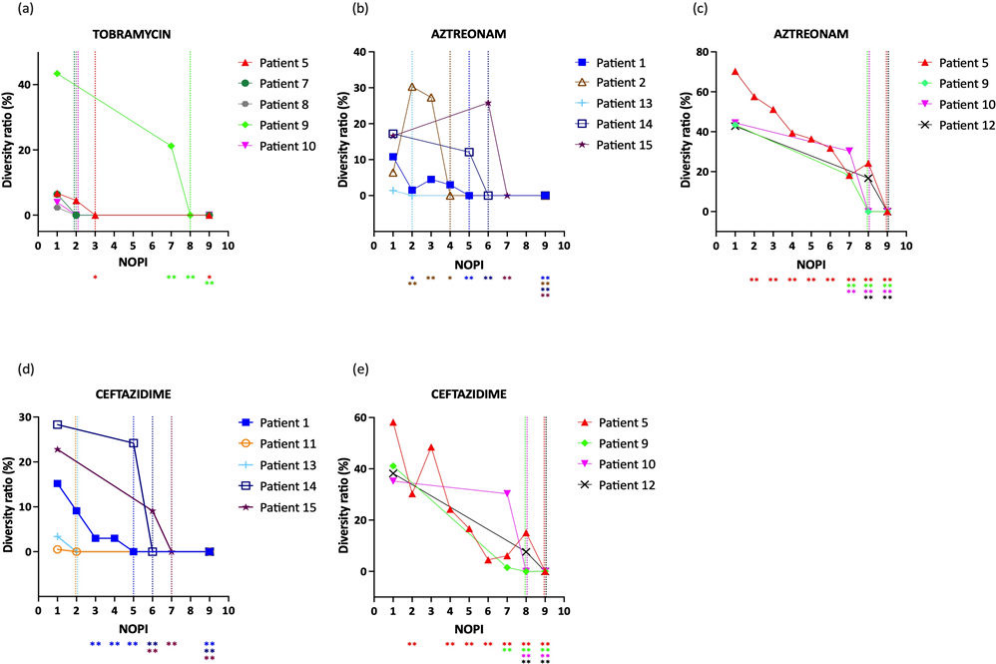
The effect of pooling isolates on the diversity ratio. The diversity ratio is depicted on the y-axis, and the number of pooled isolates (NOPI), on the x-axis. Results are shown for (**A**) tobramycin, (**B**) aztreonam (**P1, P2, P13, P14, P15**), (**C**) aztreonam (**P5, P9, P10, P2**), (**D**) ceftazidime (**P1, P11, P13, P14, P15**), and (**E**) ceftazidime (**P5, P9, P10, P12**). Data for aztreonam and ceftazidime were split in two graphs each for clarity. Dashed lines indicate the minimal number of pooled isolates for consistent MIC (mNOPI) for each patient in the corresponding color. The Fisher’s exact test was used to assess the difference in diversity ratio between single isolate and pooled isolate (i.e., two to nine isolates) testing and a Benjamini-Hochberg correction for multiple testing was performed (*P < 0.05; **P < 0.01).

The influence of pooling isolates on MIC diversity was assessed in a systematic way by pooling an increasing number of isolates and determining the diversity ratio. Pooling an increasing number of isolates resulted in a decrease in diversity ratio for all diverse MIC profiles (Fig. 3) (the only exceptions being patients 2 and 15 for which an initial increase in diversity ratio was observed for aztreonam when the number of pooled isolates was below the minimal number of pooled isolates for consistent MIC). A consistent MIC was obtained for all diverse MIC profiles after pooling two to nine isolates, i.e., for all samples and for all antibiotics, the minimal number of pooled isolates for consistent MIC varied between two and nine isolates.

**Fig 4:**
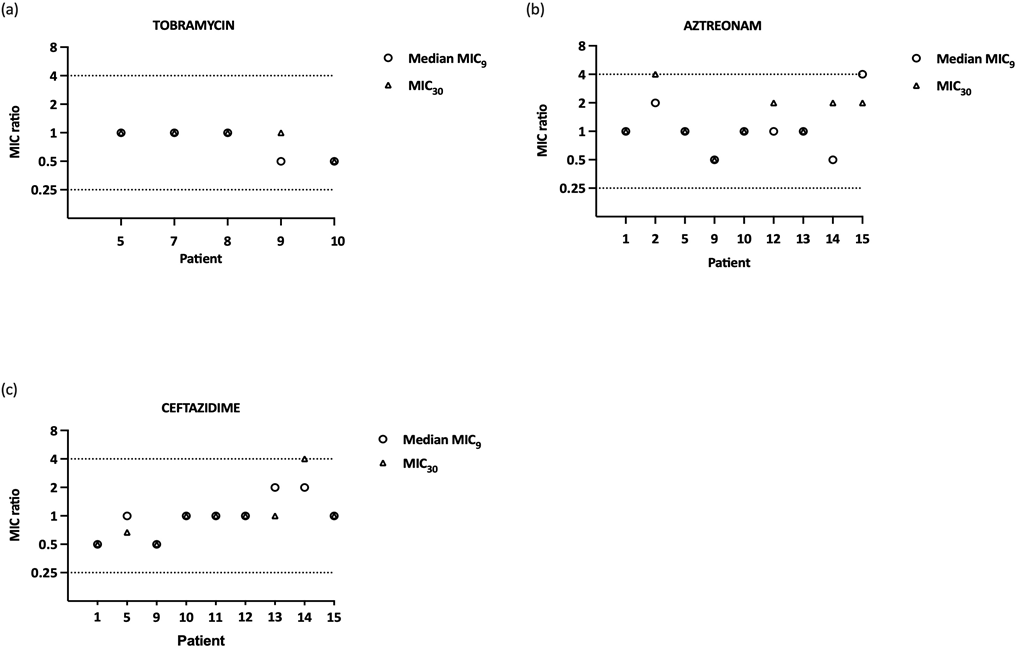
The effect of increasing the number of pooled isolates above the minimal number of pooled isolates for consistent MIC (mNOPI) for (**A**) tobramycin, (**B**) aztreonam, and (**C**) ceftazidime. The MIC ratio (depicted on the y-axis) represents the ratio of the median MIC value when nine isolates are pooled (median MIC_9_) or the MIC value of 30 pooled isolates (MIC_30_) to the median MIC value when the mNOPI is pooled. The patient number is depicted on the x-axis. The dotted lines (y = 4 and y = 0.25) show that the ratio of both the MIC_9_ and MIC_30_ to the mNOPI equaled or was lower than the acceptable fourfold change difference, indicating that increasing the number of pooled isolates above the mNOPI did not influence the MIC outcome.

Next, we evaluated whether further increasing the number of pooled isolates after reaching a consistent MIC had an influence on the diversity ratio and the MIC value. To this end, for patient 1 and the antibiotic ceftazidime, we increased the number of pooled isolates stepwise to 9 after reaching the minimal number of pooled isolates for consistent MIC (i.e., five isolates) (Fig. S1). We observed that once a consistent MIC was obtained, further increasing the number of pooled isolates did not influence the MIC anymore. Additionally, as the highest minimal number of pooled isolates for consistent MIC observed for the patient cohort was 9, the diversity ratio when pooling nine isolates was determined for all diverse MIC profiles. In all cases, pooling nine isolates led to a consistent MIC (Fig. 3). Lastly, the MIC when all 30 isolates were pooled (MIC_30_) was also determined for all diverse MIC profiles. Then, the ratio between the median MIC_9_ (median MIC value of the set of 12 combinations of 9 pooled isolates) or the MIC_30_, on the one hand, and the median MIC value of the 12 combinations of the minimal number of pooled isolates for consistent MIC (median MIC_mNOPI_), on the other hand, was determined (Fig. 4). The ratio of both the median MIC_9_ and the MIC_30_ to the median MIC_mNOPI_ never exceeded the acceptable fourfold change difference. Combined, these data indicate that using a NOPI higher than the minimal number of pooled isolates for consistent MIC does not affect the diversity ratio or MIC value.

Finally, the influence of pooling isolates for non-diverse MIC profiles was assessed by determining the MIC_30_ and the MIC for 12 combinations of 2 to 5 isolates (i.e., the minimal number of pooled isolates for consistent MIC for ceftazidime and aztreonam for this sample) for tobramycin for patient 1. For this non-diverse MIC profile, pooling isolates had no influence on the MIC (Fig. S2).

### The minimal number of pooled isolates necessary to obtain a consistent MIC depends on the MIC diversity of single isolates

**Fig 5:**
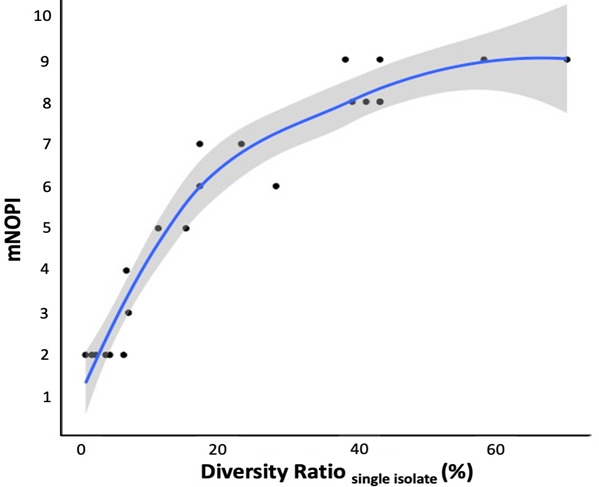
The locally estimated scatterplot smoothing (LOESS) of the relationship between the minimal number of pooled isolates for consistent MIC (mNOPI) and the diversity ratio in single isolate testing of MIC profiles with diversity. The Spearman correlation coefficient is 0.950 (*P* < 0.001; *n* = 23).

A possible explanation for the observed differences between patients and antibiotics in the minimal number of pooled isolates for consistent MIC (Fig. 3) could be the variety in diversity ratio in single isolate testing (Fig. 2). The correlation between the diversity ratio of single isolates and the minimal number of pooled isolates for consistent MIC was further investigated for all diverse MIC profiles. A statistically significant (*P* < 0.001) and strong correlation (ρ = 0.950) was observed between both variables (Fig. 5). The diversity ratio of single isolate testing can thus be considered predictive of the minimal number of pooled isolates for consistent MIC.

### MIC diversity is associated with CD

In the previous sections, we considered intrasample diversity based on MIC values. Nevertheless, MIC values are currently still interpreted based on clinical breakpoints (although minimally relevant for CF) (21), hence analyzing the findings of this study with this approach is thus of interest as well. In Fig. 2, the clinical breakpoints according to EUCAST (v 12.0) are depicted in red, visualizing the influence of diversity on the classification of isolates into EUCAST susceptibility categories. CD is observed when the MIC of one isolate compared to (an)other(s) results in classification into a different EUCAST susceptibility category (e.g., resistant vs susceptible, increased exposure). An odds ratio of 15.6 to observe CD in diverse MIC profiles, compared to non-diverse MIC profiles (*P* < 0.001; confidence interval [CI] = 2.9–83.3), was found, indicating a clear association between diversity and CD.

### Pooling isolates decreases categorical disagreement (%CD) and influences the frequency of resistance detection in a patient-specific manner

The influence of performing AST using pooled versus single isolates on clinical interpretation was studied by considering the (i) categorical disagreement and (ii) frequency of detecting antibiotic resistance.

**Fig 6:**
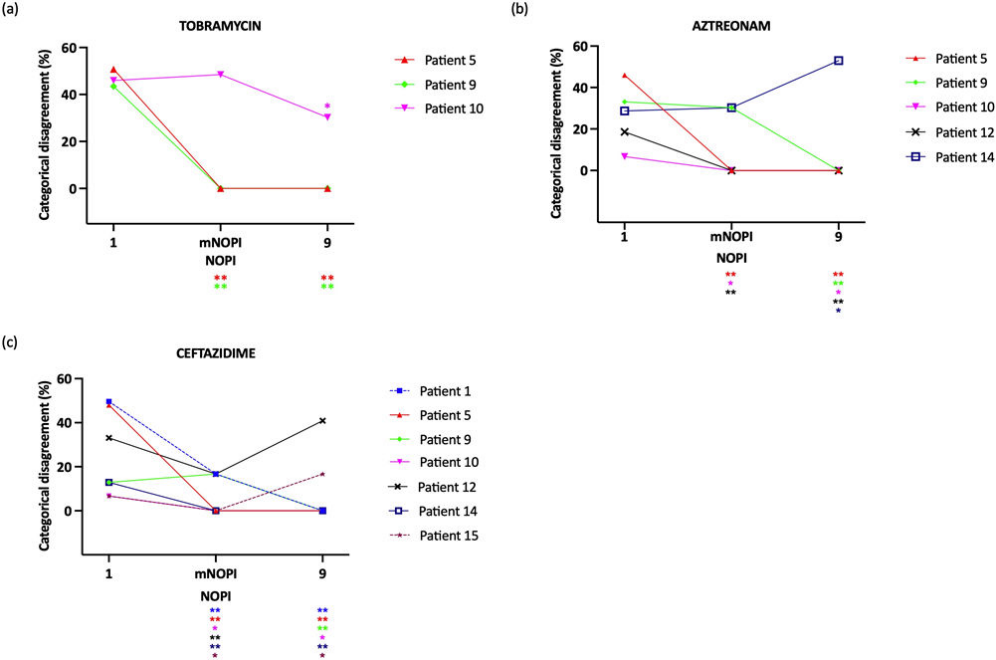
The impact of pooling isolates on the %CD for (**A**) tobramycin, (**B**) aztreonam, and (**C**) ceftazidime. Results are shown for single isolate testing and for pooled isolate testing of the minimal number of pooled isolates for consistent MIC (mNOPI) and for nine isolates. Fisher’s exact test was used to assess the difference in categorical disagreement between single isolate testing and pooled isolate testing, and a Benjamini-Hochberg correction for multiple testing was performed (*P < 0.05; **P < 0.01).

First, to evaluate the influence of pooling on categorical disagreement, when CD was observed in single isolate testing (i.e., three samples for tobramycin, five samples for aztreonam, and seven samples for ceftazidime), the %CD in single isolate testing was compared to (i) pooled isolate testing using the minimal number of pooled isolates for consistent MIC and (ii) pooled isolate testing using nine isolates (Fig. 6). The higher the %CD is, the more pairs of isolates are present in the sample, which are classified in different susceptibility categories. A significant decrease in %CD was observed after pooling the minimal number of pooled isolates for consistent MIC for 11/15 (73%) MIC profiles (tobramycin: 2/3 samples, aztreonam: 3/5 samples, ceftazidime: 6/7 samples). When pooling 9 isolates, 12/15 (80%) MIC profiles showed a significant decrease in %CD (tobramycin: 3/3 samples, aztreonam: 4/5 samples, ceftazidime: 5/7 samples). Remarkably, for ceftazidime and aztreonam, a significant increase in %CD can be observed in three samples when nine isolates were pooled, as compared to single isolate testing. This may be explained by the small proportion of isolates classified in a different susceptibility category for single isolate testing (Fig. 2), leading to a low %CD. Pooling isolates led not only to a decrease in diversity (Fig. 3) but also to a shift of the MIC closer to the resistant clinical breakpoint.

**Fig 7:**
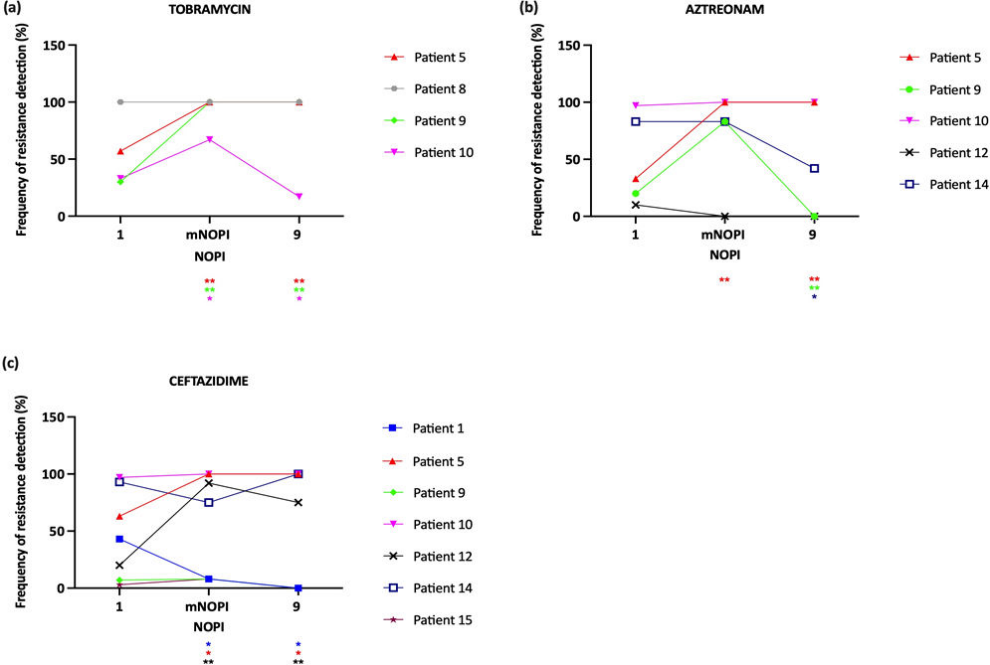
The impact of pooling isolates on the frequency of resistance detection for (**A**) tobramycin, (**B**) aztreonam, and (**C**) ceftazidime. Results are shown for single isolate testing and for pooled isolate testing of the minimal number of pooled isolates for consistent MIC (mNOPI) and for nine isolates. Fisher’s exact test was used to assess the difference in frequency of resistance detection between single isolate testing and pooled isolate testing, and a Benjamini-Hochberg correction for multiple testing was performed (*P < 0.05; **P < 0.01).

Second, the frequency of resistance detection was compared in single isolates testing and in pooled isolate testing of the minimal number of pooled isolates for consistent MIC or nine isolates (Fig. 7). If no isolates were resistant in single isolate testing, no combinations in pooled isolate testing were found to be resistant as well (data not shown). For samples that contained at least one resistant isolate in single isolate testing for a specific antibiotic, the frequency of resistance detection did not significantly decrease compared to single isolate testing, when pooling the minimal number of pooled isolates for consistent MIC for 14/16 MIC profiles (tobramycin: 4/4 samples, aztreonam: 4/5 samples, ceftazidime: 6/7 samples) (Fig. 7). When pooling nine isolates, the frequency of resistance detection did not significantly decrease compared to single isolate testing for 10/16 MIC profiles (tobramycin: 3/4 samples, aztreonam: 3/5 samples, ceftazidime: 4/7 samples) (Fig. 7). In conclusion, the frequency of resistance detection is not lower when isolates are pooled in most samples.

## DISCUSSION

*P. aeruginosa* is a highly adaptable pathogen (22), and various genetic modifications result in the occurrence of different phenotypes of the same clone in one sputum sample (22 – 24). The latter is referred to as phenotypic diversity and has been reported for different features of *P. aeruginosa*, including mucoidy, pyocyanin production, and antibiotic susceptibility (7, 22). Hence, performing standardized AST on a single isolate will often not provide a representative MIC result for the *P. aeruginosa* population present in a sample (2, 5).

In the present study, we observed intrasample MIC diversity of *P. aeruginosa* to tobramycin, aztreonam, and/or ceftazidime in 80% of patient samples investigated (13/15 samples). Diversity was most frequently observed for the tested β-lactam antibiotics (aztreonam and ceftazidime), while it was less frequent for tobramycin. In line with our results, previous studies that focused on phenotypic diversity of *P. aeruginosa* in a single patient reported diversity in antibiotic susceptibility to aztreonam, ceftazidime, and tobramycin (9 – 11, 25). Importantly, these studies all defined diversity based on categorical disagreement, rather than on absolute MIC values. Interpreting diversity in antibiotic susceptibility based on MIC values, rather than on categorical disagreement, has the advantage of being independent of clinical breakpoints, which are currently not adjusted for chronic airway infections in CF (21). Indeed, clinically achievable systemic concentrations are used to establish clinical breakpoints and have been reported to be a 100-fold lower than sputum antibiotic concentrations after inhalation of tobramycin (26). Also, defining diversity based on MIC takes into account technical and biological variation (i.e., fold change equal or lower to four in the present study). In contrast, when defining diversity based on categorical disagreement, a difference can already be observed at an MIC ratio of 2 if the MIC value is near the clinical breakpoint.

We found a meaningful correlation between the infection time with *P. aeruginosa* and MIC diversity for the tested β-lactam antibiotics, which is in line with the hypothesis that diversity occurs due to the genetic adaptation of *P. aeruginosa* to the CF airways during chronic infection (27). In a previous study, diversity in antibiotic resistance was linked to use of antibiotics belonging to the same class (5). Others claim that phenotypes change in an unpredictable way, independent of antibiotic therapy (14). In the present study, due to the cross-sectional design and the limited number of sputum samples included, no correlation could be found between antibiotic use and the occurrence of MIC diversity.

For diverse MIC profiles, the influence of pooling isolates on the MIC was systematically assessed by increasing the NOPI progressively until reaching a consistent MIC. Pooling an increasing number of isolates led to a decrease in diversity ratio in the vast majority of samples and for all antibiotics. For all diverse MIC profiles, the minimal number of pooled isolates for consistent MIC fluctuated between two and nine isolates, and a strong correlation was found between the diversity ratio of single isolates and the minimal number of pooled isolates for consistent MIC. Pooling nine isolates (i.e., the highest minimal number of pooled isolates for consistent MIC found in our study) was assessed for all diverse MIC profiles and completely abolished the MIC diversity. Extending the patient population could result in encountering patient samples with a diversity ratio that falls outside of the observed range in the present study (i.e., 0%–70%) and thus in the necessity of pooling more than nine isolates for a consistent MIC. Although the impact of extending the patient population remains to be investigated, the locally estimated scatterplot smoothing (LOESS) curve that describes the correlation between the diversity ratio in single isolate testing and the minimal number of pooled isolates suggests that only a limited increase in the minimal number of pooled isolates should be observed for patient samples with a diversity ratio above 70% to obtain a consistent MIC. We would also like to point out that sputum samples were analyzed from patients in a stable phase of the disease, where the diversity of *P. aeruginosa* may potentially be higher as compared to the diversity during an exacerbation (for example, due to the dominance of a particular phenotype and a high bacterial load). Hence, it remains to be determined whether the obtained results on the diversity of *P. aeruginosa* and pooling of isolates to generate a consistent MIC are applicable for patients undergoing an exacerbation.

To illustrate the potential impact of our findings on current clinical practice, which relies on classification in susceptibility categories, we assessed if MIC outcomes of pooled versus single isolates might influence clinical interpretation based on EUCAST (v 12.0) clinical breakpoints. To this end, CD was assessed to quantify how many isolates or combinations are classified in different susceptibility category in single versus pooled isolate testing. A clear association between MIC diversity and CD was observed, and the %CD significantly decreased in most MIC profiles when nine isolates were pooled, again highlighting that pooling isolates leads to more consistent AST results. It should be noted that some of the variation in AST occurred at high antibiotic concentrations, which may not have an impact on clinical decision making using the current breakpoints. Nevertheless, since clinical breakpoints are unadjusted to the CF population, it remains to be determined whether variation in the high antibiotic concentration range may or may not be clinically meaningful for CF patients.

Second, we assessed if pooling isolates led to a lower or higher frequency of resistance detection. In contrast to Foweraker et al. who reported resistance, observed in single isolate testing, to be missed when pooling four isolates in 75% of the samples (45/60 samples) (13), we detected resistance as frequent or more frequent in pooled isolate testing in most patient samples (88% when pooling the minimal number of pooled isolates for consistent MIC, 63% when pooling nine isolates). However, based on our results, it is not possible to predict whether pooling will lead to more or less frequent detection of resistance, which is probably a consequence of a complex interplay between *P. aeruginosa* phenotypes (with, e.g., different growth rates, resistance mechanisms). Also, it should be noted that the link between resistance (based on single isolate testing) and treatment efficacy in CF remains unclear, as a previous study reported successful treatment with tobramycin despite the presence of tobramycin-resistant *P. aeruginosa* isolates (3, 28).

In the present study, we provide evidence that pooling *P. aeruginosa* isolates is a promising approach to obtain more consistent MIC results for CF. Clinical studies are now required to evaluate whether the MIC of pooled isolates is more predictive of an efficacious treatment. If so, this could open new perspectives for AST in pwCF. In addition to considering phenotypic diversity of *P. aeruginosa* when performing AST, it should be pointed out that *P. aeruginosa* appears as biofilm-like microcolonies in the CF lung, which are associated with higher antibiotic tolerance and are not considered in standardized AST on planktonic bacteria (4). Also, other factors of the CF lung microenvironment (host factors and microbiota) have been shown to influence the antibiotic susceptibility of this pathogen, which may contribute to the discrepancy between *in vitro* and *in vivo* antibiotic efficacy (27) and may need to be considered as well in the quest for more predictive AST in the CF population. An important limitation of this study design is, therefore, that we could not consider the influence of clinical factors such as the use of inhaled antibiotics, oral macrolides, and/or CFTR modulators and microbiological factors such as other microorganisms co-infecting the airway on AST outcome.

The results of the present study can possibly be extended to other CF pathogens and even to other infectious diseases for which intrasample phenotypic diversity was described. Indeed, intrasample diversity in antibiotic susceptibility was also reported for *Staphylococcus aureus* and *Achromobacter xylosoxidans* in CF (29 – 31). In addition, phenotypic diversity is not limited to airway infectious diseases but was also found in extra-intestinal infections by *Escherichia coli* and gastric infections by *Helicobacter pylori* (32, 33).

## SUPPLEMENTAL MATERIAL

Supplemental Material spectrum.00449-23-s0001.pdf Supplementary methods and results.

ASM does not own the copyrights to Supplemental Material that may be linked to, or accessed through, an article. The authors have granted ASM a non-exclusive, world-wide license to publish the Supplemental Material files. Please contact the corresponding author directly for reuse.
