# Supplementary material for: Pooling isolates to address the diversity in antimicrobial susceptibility of Pseudomonas aeruginosa in cystic fibrosis
Source: Microbiol Spectr. 2023 Nov 20;11(6):e00449-23. doi: 10.1128/spectrum.00449-23 (PMC10714813; doi:10.1128/spectrum.00449-23)
Supplement: Supplemental Material — Supplementary methods and results. [file spectrum.00449-23-s0001.pdf]

SUPPLEMENTARY DATA

Table S1. Patient characteristics.

Number	Date of sampling	Sex	Age	Genotype	CFTR modulator treatment*	FEV1** (% predicted)	Pancreatic insufficiency	Infection time with *P. aeruginosa* (years)	Other isolated microorganisms°	Antibiotic use at time of sampling	Antibiotic use in the year preceding the sampling		
											Intravenous antibiotics	Inhaled antibiotics°°°	Oral antibiotics
1	11/09/2019	Male	36	F/other	IVA	102	Yes	> 17	*Haemophilus parainfluenzae, Serratia sp.*	Clindamycin	None	None	Ciprofloxacin, clindamycin
2	25/09/2019	Male	28	F/other	None	52	Yes	2	*Aspergillus fumigatus, Haemophilus influenzae, Staphylococcus aureus*	None	None	Tobramycin	None
3	16/10/2019	Male	20	F/F	None	64	Yes	1	*Haemophilus parahaemolyticus, Staphylococcus aureus, Stenotrophomonas maltophilia*	Azithromycin***, ciprofloxacin°°	Ceftazidime, piperacillin-tazobactam, tobramycin	Colistimethate°°	Azithromycin***, sulfamethoxazole-trimethoprim
4	23/10/2019	Female	20	F/F	None	36	Yes	14	*Aspergillus flavus, Aspergillus fumigatus, Mycobacterium intracellulare, Staphylococcus aureus, Stenotrophomonas maltophilia*	Azithromycin, clofazimine, ethambutol, inhaled colistimethate, rifabutin	Amikacin, ceftazidime, piperacillin-tazobactam, tobramycin	Aztreonam, colistimethate	Azithromycin, clofazimine, ethambutol, inhaled colistimethate, rifabutin
5	19/02/2020	Female	28	F/F	None	65	Yes	> 19	*Achromobacter xylosoxidans, Haemophilus parainfluenzae, Staphylococcus aureus*	Azithromycin***, inhaled colistimethate	Colistimethate, meropenem, piperacillin-tazobactam, tobramycin	Aztreonam, colistimethate	Amoxiclav, Azithromycin***, ciprofloxacin, sulfamethoxazole-trimethoprim
6	14/10/2020	Male	21	F/F	None	71	Yes	1	*Aspergillus fumigatus, Mycobacterium intracellulare, Stenotrophomonas maltophilia, Streptococcus pneumoniae*	None	Colistimethate, piperacillin-tazobactam, tobramycin	Colistimethate	Ciprofloxacin, sulfamethoxazole-trimethoprim
7	14/10/2020	Female	18	F/F	LUM/IVA	74	Yes	1	*Staphylococcus aureus, Streptococcus agalactiae*	Inhaled aztreonam	Piperacillin-tazobactam, tobramycin	Aztreonam, colistimethate	Amoxiclav, ciprofloxacin

F: *Phe508del*, other = other CF-causing mutation than *Phe508del*, IVA: ivacaftor, LUM: lumacaftor, TEZ: tezacaftor, ELX: elexacftor, PA: *Pseudomonas aeruginosa*
treatment at timing of sampling, **measurement at time of sampling, best value, after bronchodilatation if applicable, ***immunomodulatory dose, °up to one year before sampling, °°*Pseudomonas aeruginosa* eradication regimen, °°°when two antibiotics are mentioned, these are monthly alternated, ‡chronic suppression therapy for *Achromobacter xylosoxidans

											Antibiotic use in the year preceding the sampling		
**Number**	Date of sampling	Sex	Age	Genotype	CFTR modulator treatment*	FEV1** (% predicted)	Pancreatic insufficiency	Infection time with *P. aeruginosa* (years)	Other isolated microorganisms°	Antibiotic use at time of sampling	Intravenous antibiotics	Inhaled antibiotics°°°	Oral antibiotics
**8**	21/10/2020	Female	59	F/other	ELX/TEZ/IVA	35	Yes	1	*Staphylococcus aureus*	Azithromycin***	Ceftazidime, colistimethate, meropenem, piperacillin-tazobactam, tobramycin	Aztreonam	Azithromycin***, ciprofloxacin
**9**	13/01/2021	Female	34	other/other	None	19	Yes	> 12	*Aspergillus fumigatus, Haemophilus parainfluenzae, Staphylococcus aureus, Serratia marcescens*	Azithromycin***, inhaled colistimethate	Colistimethate, meropenem	Colistimethate	Amoxiclav, azithromycin***, ciprofloxacin
**10**	10/02/2021	Female	27	F/other	None	49	Yes	> 19	*Staphylococcus aureus*	Inhaled aztreonam	Ceftazidime, piperacillin-tazobactam, tobramycin	Aztreonam	Ciprofloxacin, flucloxacilline, sulfamethoxazole-trimethoprim
**11**	24/02/2021	Male	25	other/other	None	57	Yes	6	*Achromobacter xylosoxidans*	Inhaled colistimethate, minocyclin[#], roxithromycin***, sulfamethoxazole-trimethoprim[#]	Colistimethate, meropenem	Aztreonam, colistimethate	Ciprofloxacin, minocyclin[#], roxithromycin***, sulfamethoxazole-trimethoprim[#]
**12**	06/05/2021	Female	41	F/F	None	61	Yes	> 19	*Aspergillus fumigatus*	Azithromycin***, inhaled aztreonam	None	Aztreonam, colistimethate	Azithromycin***
**13**	03/06/2021	Female	33	other/other	None	76	Yes	> 19	*Haemophilus parainfluenzae, Klebsiella oxytoca, Staphylococcus aureus*	Azithromycin***	Ceftazidime, piperacillin-tazobactam, tobramycin	None	Azithromycin***, ciprofloxacin
**14**	21/06/2021	Female	35	F/F	TEZ/IVA	47	Yes	19	*Aspergillus fumigatus, Stenotrophomonas maltophilia*	Azithromycin***, inhaled aztreonam	Ceftazidime, tobramycin	Aztreonam, colistimethate	Azithromycin***, ciprofloxacin
**15**	11/08/2021	Male	41	F/other	IVA	64	Yes	19	*Haemophilus parainfluenzae*	None	None	None	Ciprofloxacin

F: *Phe508del*, other = other CF-causing mutation than *Phe508del*, IVA: ivacaftor, LUM: lumacaftor, TEZ: tezacaftor, ELX: elexacftor, PA: *Pseudomonas aeruginosa*
treatment at timing of sampling, **measurement at time of sampling, best value, after bronchodilatation if applicable, ***immunomodulatory dose, °up to one year before sampling, °°*Pseudomonas aeruginosa* eradication regimen, °°°when two antibiotics are mentioned, these are monthly alternated, [#]chronic suppression therapy for *Achromobacter xylosoxidans

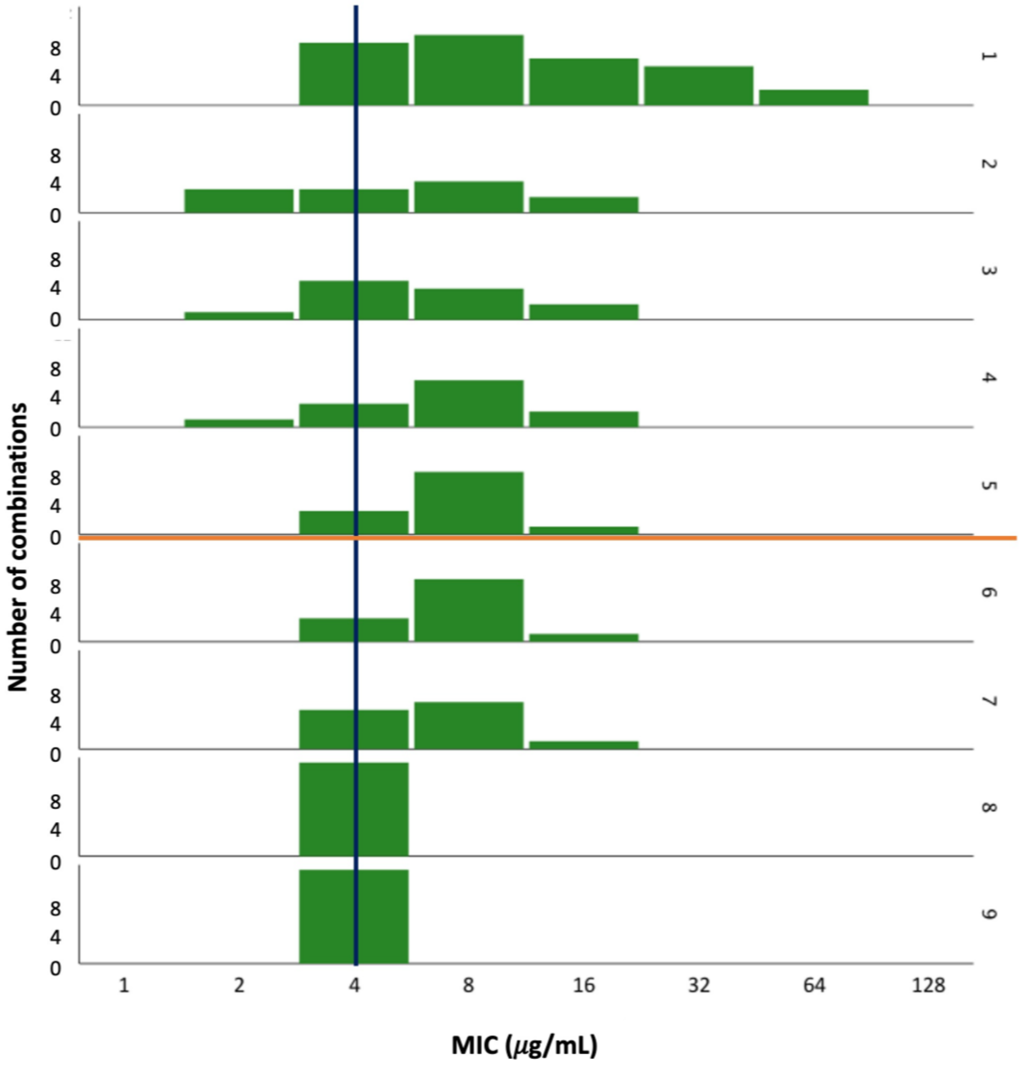

Figure S1. Histogram of MIC values for single isolate testing and for pooled isolate testing of 2-9 isolates for patient 1 are shown for ceftazidime. On the horizontal axis the MIC value is depicted, on the vertical axis the frequency (i.e. number of isolates or combinations with that particular MIC) is shown for different numbers of pooled isolates (number of pooled isolates shown on the right). The MIC value for the combination of all 30 isolates (MIC_{30}) is depicted in blue (4 μg/mL). The orange line represents the minimal number of pooled isolates necessary to obtain a consistent MIC (minimal number of pooled isolates for consistent MIC = 5).

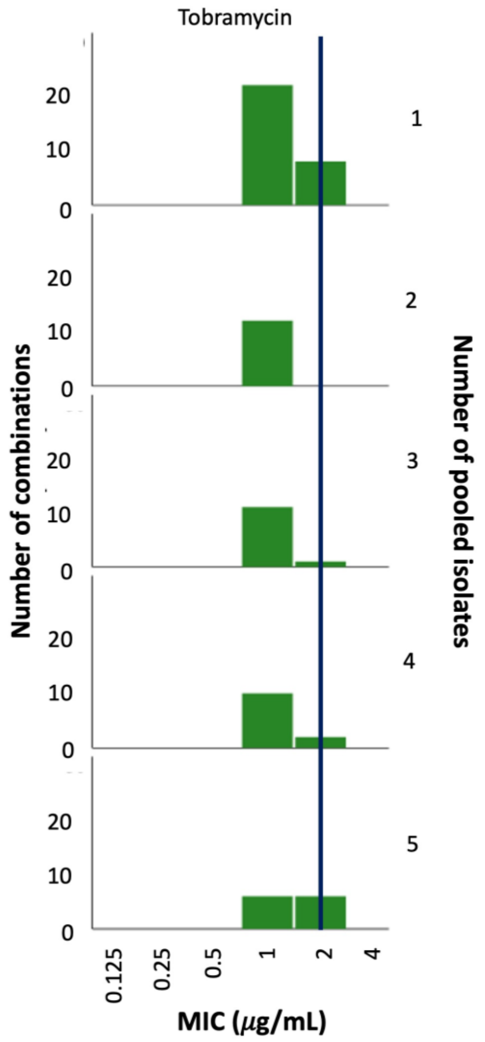

Figure S2. Histogram of MIC values for single isolate testing and for pooled isolate testing of 2-5 isolates for patient 1 are shown for tobramycin. On the horizontal axis the MIC value is depicted, on the vertical axis the frequency (i.e. number of isolates or combinations with that particular MIC) is shown for different numbers of pooled isolates (number of pooled isolates shown on the right). The MIC values for the combination of all 30 isolates for tobramycin (MIC_{30}) is depicted in blue (2 μg/mL).
